# Identifying potential biomarkers for the diagnosis and treatment of IgA nephropathy based on bioinformatics analysis

**DOI:** 10.1186/s12920-023-01494-y

**Published:** 2023-03-28

**Authors:** Xiaohui Li, Mengru Zeng, Jialu Liu, Shumin Zhang, Yifei Liu, Yuee Zhao, Cong Wei, Kexin Yang, Ying Huang, Lei Zhang, Li Xiao

**Affiliations:** Department of Nephrology, Hunan Key Laboratory of Kidney Disease and Blood Purification, The Second Xiangya Hospital, Central South University, Changsha, 410011 China

**Keywords:** IgA nephropathy, Bioinformatics analysis, Hub genes, Diagnosis, Therapeutics, Biomarkers

## Abstract

**Background:**

IgA nephropathy (IgAN) has become the leading cause of end-stage renal disease in young adults. Nevertheless, the current diagnosis exclusively relies on invasive renal biopsy, and specific treatment is deficient. Thus, our study aims to identify potential crucial genes, thereby providing novel biomarkers for the diagnosis and therapy of IgAN.

**Methods:**

Three microarray datasets were downloaded from GEO official website. Differentially expressed genes (DEGs) were identified by limma package. GO and KEGG analysis were conducted. Tissue/organ-specific DEGs were distinguished via BioGPS. GSEA was utilized to elucidate the predominant enrichment pathways. The PPI network of DEGs was established, and hub genes were mined through Cytoscape. The CTD database was employed to determine the association between hub genes and IgAN. Infiltrating immune cells and their relationship to hub genes were evaluated based on CIBERSORT. Furthermore, the diagnostic effectiveness of hub markers was subsequently predicted using the ROC curves. The CMap database was applied to investigate potential therapeutic drugs. The expression level and diagnostic accuracy of TYROBP was validated in the cell model of IgAN and different renal pathologies.

**Results:**

A total of 113 DEGs were screened, which were mostly enriched in peptidase regulator activity, regulation of cytokine production, and collagen-containing extracellular matrix. Among these DEGs, 67 genes manifested pronounced tissue and organ specificity. GSEA analysis revealed that the most significant enriched gene sets were involved in proteasome pathway. Ten hub genes (KNG1, FN1, ALB, PLG, IGF1, EGF, HRG, TYROBP, CSF1R, and ITGB2) were recognized. CTD showed a close connection between ALB, IGF, FN1 and IgAN. Immune infiltration analysis elucidated that IGF1, EGF, HRG, FN1, ITGB2, and TYROBP were closely associated with infiltrating immune cells. ROC curves reflected that all hub genes, especially TYROBP, exhibited a good diagnostic value for IgAN. Verteporfin, moxonidine, and procaine were the most significant three therapeutic drugs. Further exploration proved that TYROBP was not only highly expressed in IgAN, but exhibited high specificity for the diagnosis of IgAN.

**Conclusions:**

This study may offer novel insights into the mechanisms involved in IgAN occurrence and progression and the selection of diagnostic markers and therapeutic targets for IgAN.

**Supplementary Information:**

The online version contains supplementary material available at 10.1186/s12920-023-01494-y.

## Introduction

IgA nephropathy (IgAN) is recognized as the most prevalent primary glomerular disease worldwide accompanied by a global incidence rate of more than 2.5 per 100,000 [1, 2]. In addition, IgAN has accounted for the biggest proportion of end-stage renal disease (ESRD) in the young with up to 30% of IgAN ultimately ending up as ESRD within 20 years and demanding renal replacement therapy [3, 4]. Clinically, the common manifestation of IgAN is macroscopic hematuria which frequently follows a mucosal infection, such as an upper respiratory or gastrointestinal infection [1]. However, the heterogeneity of clinical course is so substantial that patients with IgAN may also present highly diverse syndromes ranging from minor urinary abnormalities to rapidly progressive glomerulonephritis [5]. Due to the lack of specific biomarkers, the current gold standard for diagnosis of IgAN exclusively relies on renal biopsy characterized by dominant IgA deposition in the glomerular mesangial region [1]. Among multitudinous concepts regarding the pathogenesis of IgAN, the four-hit theory is widely accepted: excessive abnormal hypogalactosylated IgA1 (Gd-IgA1) are induced by unknown factors and serve as antigen leading to the production of specific autoantibodies, subsequent antigen-antibody complexes take form in the blood circulation, thus finally deposit in the glomerular mesangium [6, 7]. As a result, chronic inflammation and kidney impairment occur [8]. Still, there are a great many underlying mechanisms left unexplored. Regrettably, the main therapies including immunosuppression and supportive care, are still unsatisfactory. Given the invasive diagnosis and unclear mechanisms as well as limited therapies, identifying underlying mechanisms and key biomarkers to provide novel diagnosis and optimal treatment is warranted.

In recent years, microarray and bioinformatic analyses have been extensively employed to detect new biomarkers and potential molecular pathogenesis in various diseases but not many in IgAN. This research is committed to screening potential hub genes involved in IgAN, thereby providing novel markers for non-invasive diagnosis and potential targets for treatment of IgAN. Most previous studies applied two datasets to identify some hub genes with or without validation by external validation set. There are several innovation points of this study. Firstly, our study integrated three datasets which contained almost all human IgAN sample currently uploaded to GEO. Secondly, our study applied as many bioinformatics methods as possible to systematically and comprehensively elucidate the underlying mechanisms of IgAN. Thirdly, one hub gene with the highest expression level and the strongest diagnostic ability among identified 10 hub genes was also verified using in vitro and in vivo experiments. Consequently, three microarray data sets from Gene Expression Omnibus (GEO) database were obtained and related expression profiles were extracted. Then, this study identified differentially expressed genes (DEGs) between IgAN patients and the normal controls, and determined tissue- or organ-specific expressed genes via BioGPS. DEGs and all detected genes were subsequently exposed to Gene Ontology (GO) and Kyoto Encyclopedia of Genes and Genomes (KEGG) enrichment analysis using clusterProfiler package in R software and Gene set enrichment analysis (GSEA) software respectively. Next, protein-protein interaction (PPI) network was established based on the STRING tool, and key modules and hub genes were screened by Cytoscape. Maximal Clique Centrality (MCC) algorithm in Cytohubba was also utilized to determine the top ten hub genes. Eventually, the intersection of the two results was deemed the final hub genes, out of which several genes were found closely associated with IgAN via the Comparative Toxicogenomics Database (CTD) database. The GO and KEGG functional enrichment of these ten hub genes were investigated through ClueGO and Cluepedia tools in Cytoscape. To verify the identified hub genes, Nephroseq v5 online platform was applied. Further, ROC curve and immune infiltration analysis were performed to assess the diagnostic ability of the selected hub genes and elucidate the correlation between the selected hub genes and the immune microenvironment separately. Subsequently, our study predicted potential therapeutic drugs for IgAN via the CMap online database, followed by the chemical structure of identified small molecular drugs retrieved from the PubChem database. Ultimately, TYROBP gene with the highest levels among unexplored hub genes was selected to perform further validation. In conclusion, this study unraveled the mechanisms of disease development in IgAN and identified 10 hub genes, which may become prospective biomarkers for noninvasive diagnosis and therapeutic targets to improve the prognosis of IgAN.

## Materials and methods

### Microarray data acquisition

“IgA nephropathy” served as search term on GEO database, and “Homo sapiens” and “Expression profiling by array” were used for further screening. The microarray datasets with IgAN glomerular data were included and those with duplicate sample data were excluded. Three microarray data (GSE37460, GSE99339, and GSE104948) were screened and downloaded from GEO (https://www.ncbi.nlm.nih.gov/geo/), which is a public repository containing high-throughput genomic data, microarrays, and chips [9]. GSE37460 is based on two platforms, namely GPL11670 (Affymetrix Human Genome U133 Plus 2.0 Array) and GPL14663 (Affymetrix GeneChip Human Genome HG-U133A Custom CDF). The former platform includes eighteen glomerular tissue samples from normal controls while the latter platform includes twenty-seven glomerular tissue samples from patients with IgAN and nine normal controls [10]. GSE99339 based on GPL19184 (Affymetrix Human Genome U133A Array), contains twenty-six glomerular tissue samples from IgAN patients [11], and GSE104948 based on GPL24120 (Affymetrix Human Genome U133A Array), collects twenty-seven glomerular tissue samples from IgAN patients [12].

### Data normalization and DEGs identification

The probes in three downloaded original files were converted into gene symbols. Among them, probes without corresponding gene symbols were discarded and genes with multiple probes were averaged. After that, our study merged samples which were from IgAN and normal group in three microarray data sets. Further, the integrated data was removed batch effect by sva package in R software (version 4.0.5). Finally, the limma package was applied to detect DEGs between glomerular tissues of patients with IgAN and the controls. Genes satisfying the screening criteria of the absolute value of log FC (fold change) > 1 and p < 0.05 were judged to be statistically significant, which were visualized by heatmaps and volcano plots based on R software.

### Tissue/Organ-Specific gene expression

The online tool BioGPS (http://biogps.org) was utilized to explore tissue/organ-specific expression of the DEGs [13]. Transcripts complying the following screening standards were esteemed highly tissue-specific: [1] localized to a single organ system with the expression level of more than 10 times the median, and [2] the expression in the second-most abundant tissue was less than one-third of the highest level [14, 15].

### Functional enrichment analysis

In order to fully understand the pathogenic process DEGs involved in, clusterprofiler package in R software was employed to conduct GO and KEGG enrichment analysis of DEGs [16]. GO analysis consists of molecular function (MF), biological process (BP) and cellular component (CC), with the goal of indicating protein function [17]. KEGG was performed mainly for pathway analysis (www.kegg.jp/kegg/kegg1.html).

[18–20]. GO term and KEGG pathways with p < 0.05 and gene count > 2 were deemed significant. In addition, the study also performed KEGG enrichment analysis of all detected genes via GSEA software (version 4.1) and c2:curated gene sets (c2.cp.kegg.v7.1.symbols.gmt) [21].

### PPI network construction and hub genes recognition

The Protein-Protein Interaction (PPI) network of DEGs was established through the online tool STRING (https://string-db.org/), which predicted interactions between proteins and determined the mechanisms of IgAN [22, 23]. Interaction with a combined score > 0.7 was set as the threshold for PPI network construction, and the result was visualized based on Cytoscape software (v3.8.2) [24]. Key modules and hub genes were acquired with MCODE score ≥ 2 via Cytoscape’s plug-in molecular complex detection (MCODE). Besides, the top 10 hub genes were also defined by MCC algorithm in the plug-in cytoHubba of Cytoscape. Ultimately, the intersecting genes of the two results were recognized as the final hub genes. GO and KEGG analyses of final hub genes were then examined and showed using ClueGO and Cluepedia tools in Cytoscape.

### The association between screened hub genes and IgAN

The comparative toxicogenomics database (CTD, http://ctdbase.org) is a public database featuring abundant information regarding interactions between chemicals, genes, and diseases [25]. The CTD database was used to elucidate the relationship between hub genes and IgAN risk.

### Immune infiltration analysis

The CIBERSORT package was employed to retrieve the fraction of immune cells. The CIBERSORT algorithm is based on a predefined immune signature matrix and gene expression array to calculate the relative proportions of 22 kinds of immune cell subsets in these samples. Principal component analysis (PCA) was conducted to analyze whether immune cell infiltration in IgAN glomerulus differed from that in normal controls. The difference in each immune cell infiltration between patients with IgAN and normal samples was determined using the ggplot2 package in R language. The R software package psych was used to compute Pearson’s correlation coefficient between immune cell subpopulations and hub genes.

### Expression of hub genes grounded on nephroseq v5 online platform

The expression of hub genes in patients with IgAN and normal samples was observed using Nephroseq v5 online platform (http://v5.nephroseq.org). Group data were presented as mean ± SEM. Two-group comparisons were performed by Student’s t-test and significant difference in statistics was indicated by *p < 0.05,**p < 0.01,***p < 0.001.

### ROC curves of hub genes

To evaluate the levels of identified hub genes distinguishing IgAN from the normal group, the receiver-operating characteristic (ROC) curve analysis was employed. The R software pROC package was used for implementing data analysis and the ggplot2 package for visualizing the results.

### Small molecule therapeutic drugs prediction

The Connectivity Map (CMap) is an online database embracing the functional relationship between diseases, genes and small molecular compounds based on intervention gene expression profiles and commonly applied to predict potential drugs for diseases therapy [26, 27]. A negative connectivity score indicates the drugs may be candidates for the treatment of diseases by reversing the specific gene expression pattern in disease states. These ten hub genes were submitted to the CMap database to predict potential therapeutic drugs ameliorating IgAN prognosis. PubChem (https://pubchem.ncbi.nlm.nih.gov/) was utilized to obtain the chemical structure of identified small molecule drugs.

### Cell culture and treatment

The human glomerular mesangial cell line HMC (CBR130735, Cellbio, China) was grown in Dulbecco’s Modified Eagle’s Medium/Nutrient F-12 Ham (DMEM/F12) medium (Gibco, USA) plus10% fetal bovine serum (FBS) (BI) and 1% antibiotics at 37 °C in an atmosphere of 5% CO2. Aggregated IgA (aIgA1) were acquired by heating and aggregating monomeric human IgA1(Abcam) for 150 min at 65 °C as previously described 7. Then, HMCs were incubated with 25 µg/ml concentrations of aIgA1 for 24 h to creat the cell model of IgAN and were collected for western blot.

### Western blot method

Briefly, the protein extraction from HMC cells was conducted using radio immunoprecipitation assay (RIPA) buffer with protease/phosphatase inhibitor cocktail. Protein concentration was detected by a BCA protein assay kit (Thermo Fisher Scientific). Proteins were separated using 8–12% SDS-PAGE and later transferred onto PVDF membranes. After blocking with 5% skim milk in phosphate-buffered saline solution containing 0.1% Tween-20, the blots were exposed to the anti-TYROBP primary antibody (Santa, 1:1000). β-actin was applied as an internal reference.

### Immunohistochemistry staining

Paraffin-embedded renal sections were dewaxed and rehydrated, followed by antigen retrieval and blocking. Subsequently, the sections were incubated with the primary anti-TYROBP antibody (Santa, 1:200) overnight at 4 °C. After that, the sections were incubated with HRP-conjugated secondary antibodies and diaminobenzidine (DAB) served as a substrate to develop color. Images were obtained by a Nikon microscope and analyzed by Image-Pro plus 6.0. In the validation cohort, 15 patients were enrolled. These patients consisted of 3 minimal change disease (MCD) patients, 3 IgAN patients, 3 diabetic nephropathy (DN) patients, 3 focal segmental glomerular sclerosis (FSGS) patients and 3 membranous nephropathy (MN) patients. Immunohistochemical staining was conducted on three independent cases and controls.

## Results

### Identification of DEGs in IgAN

**Fig. 1:**
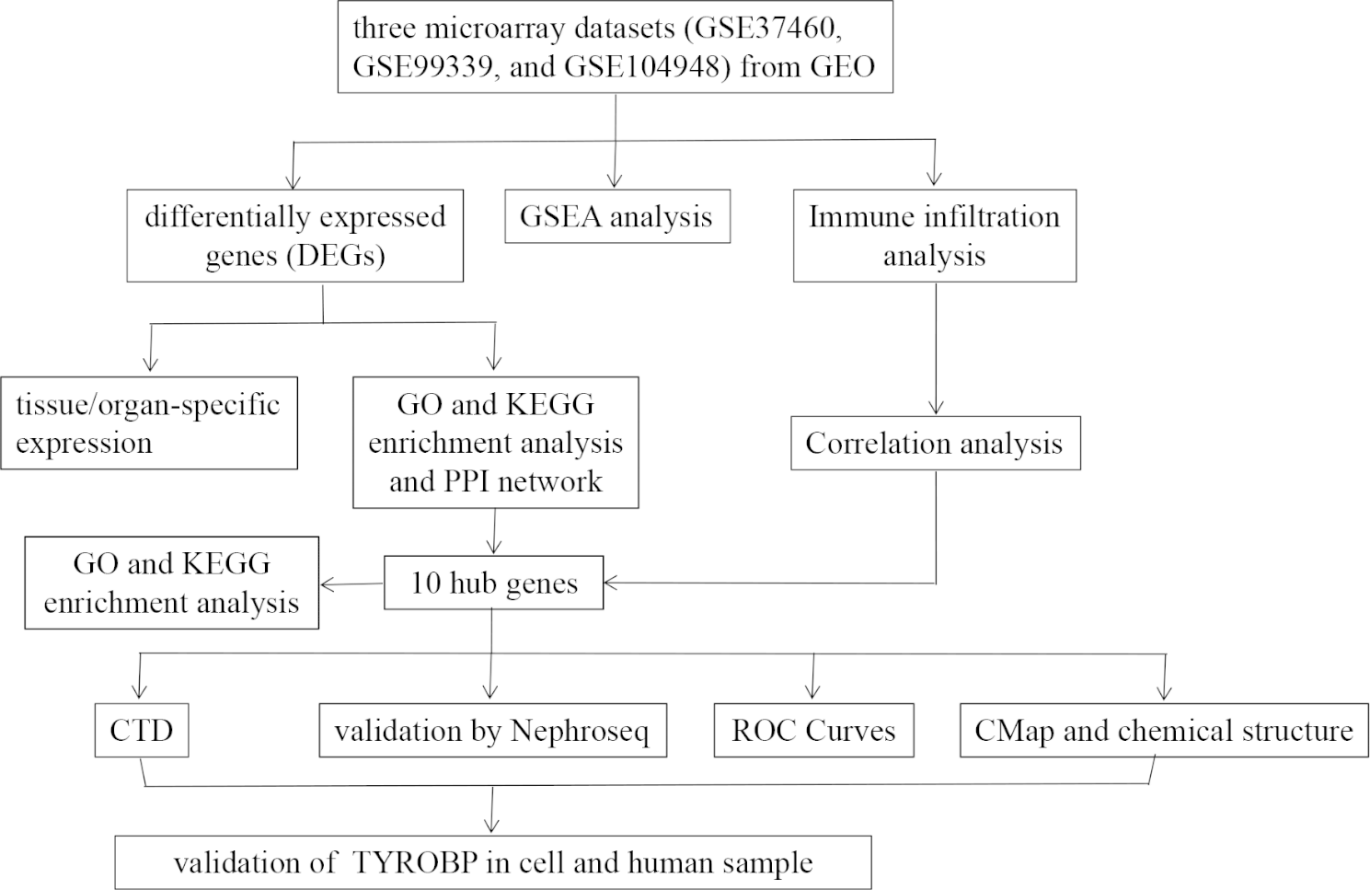
Flow chart to demonstrate the process of data analysis and experimental validation

**Fig. 2:**
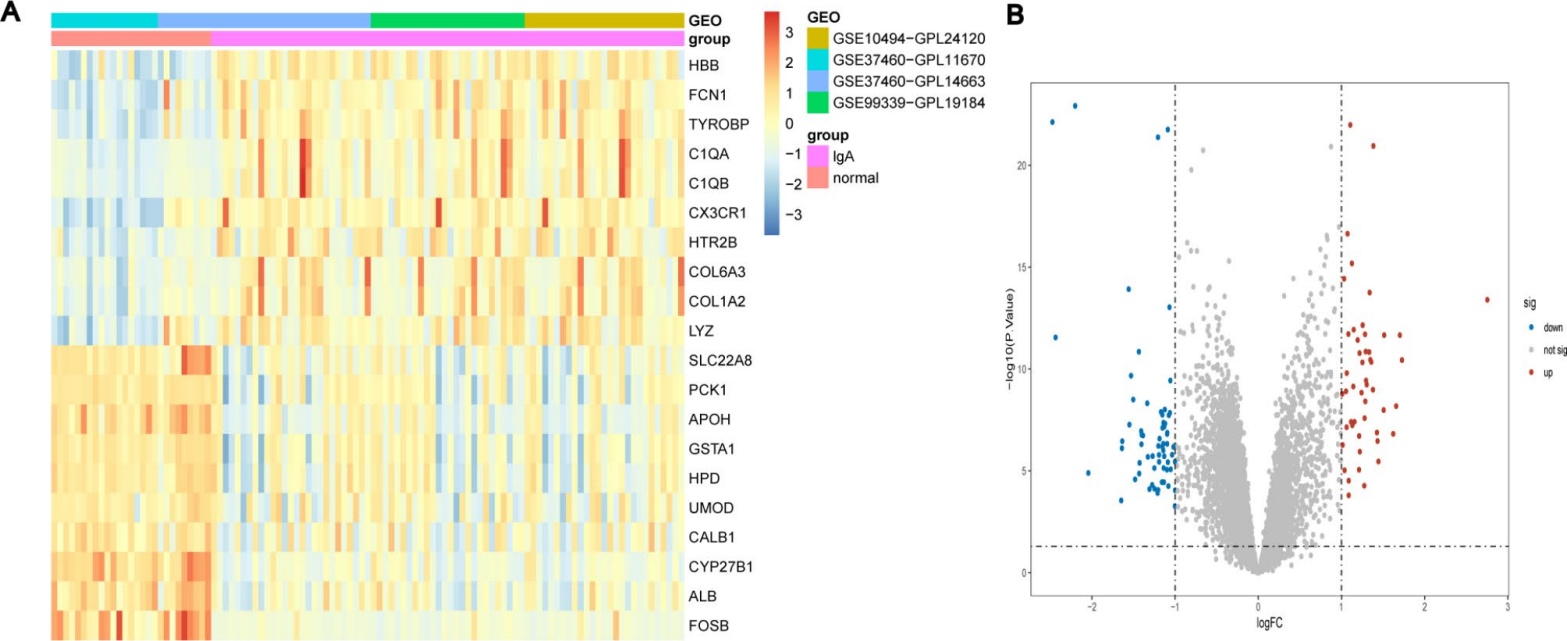
Identification of DEGs in renal glomerular tissue from IgAN patients compared with control samples samples. **(A)** Heatmap of the top 10 upregulated genes and the top 10 downregulated genes. Red rectangles indicate upregulated genes and blue rectangles indicate downregulated genes. **(B)** Volcano plot of identified DEGs. Red dots indicate upregulated genes and blue dots indicate downregulated genes. DEG: differentially expressed gene; IgAN: IgA nephropathy

This study design was depicted in Fig. 1. Three microarray datasets (GSE37460, GSE99339, and GSE104948) were downloaded to obtain DEGs related to IgAN. After normalizing the microarray results, a total of 113 DEGs involved in IgAN were filtered by limma package (p < 0.05, |logFC| > 1), including 49 up-regulated genes and 64 down-regulated genes, as shown in the heatmap and volcano plot (Fig. 2).

### Identification of tissue- or organ-specific expressed genes

This study identified 67 tissue/organ-specific expressed genes via BioGPS (Table 1). Among them, most of these genes were specifically expressed in the haematologic/immune system (27/67, 40.30%). The digestive system was the second organ-specific expressed system, which comprised 15 genes (15/67, 22.39%). It was followed by the urinary system (6/67, 8.96%), genital system (5/67, 7.46%), endocrine system (3/67, 4.48%), skeletal/muscle system (3/67, 4.48%), and placenta (2/67, 2.99%). At last, the respiratory system, circulatory system, tongue and adipose tissues shared the lowest specific expressed genes (1/67, 1.49%). However, the results also showed that 17 genes were highly expressed in both the digestive system and kidney, such as PRODH2, SLC27A2, GBA3, PBLD, PCK1, FBP1, HAO2, GLYAT, HPD, BHMT2, APOM, DPYS, GATM, SLC7A9, DPEP1, PLG, and DEFB1.

**Table 1:** Distribution of tissue/organ-specific expressed DEGs from BioGPS

System/Organ	Genes	Counts
Haematologic/Immune		
Haematologic/Immune cells	FOSB,LPAR6,HLX,GBP2,HCLS1,FGL2,LY96,FCER1G, CX3CR1,TYROBP,FCN1,HCK,PTPRC,NCF2,PYCARD, MS4A6A,MGAM,ISG15	18
Immune organs	CD48,IL10RA,CD53,ITGB2,CD52,PLAC8,LTF,CXCL14, DI01	9
Nervous	CALB1,NETO2	2
Digestive	APOH,ALB,HRG,KNG1,AFM,GBA3,HMGCS2,FTCD, SERPINA6,FABP1,UPB1,CYP4F2,RBP4,KLK1,NNMT	15
Urinary	SLC22A8,EGF,ALDH6A1,XPNPEP2,SLC13A3,SLC22A6	6
Respiratory	VSIG4	1
Circulatory	C1QB	1
Endocrine	SLC19A2,NR4A2,SST	3
Skeletal/muscle	PLK2,POSTN,TGFBI	3
Placenta	CSF1R,HSD11B2	2
Genital	IGF1,ACE2,SLC17A3,SERPINA5,UMOD	5
Others		
Tongue	ECM1	1
Adipose	CD36	1

### GO and KEGG enrichment analyses of DEGs

**Fig. 3:**
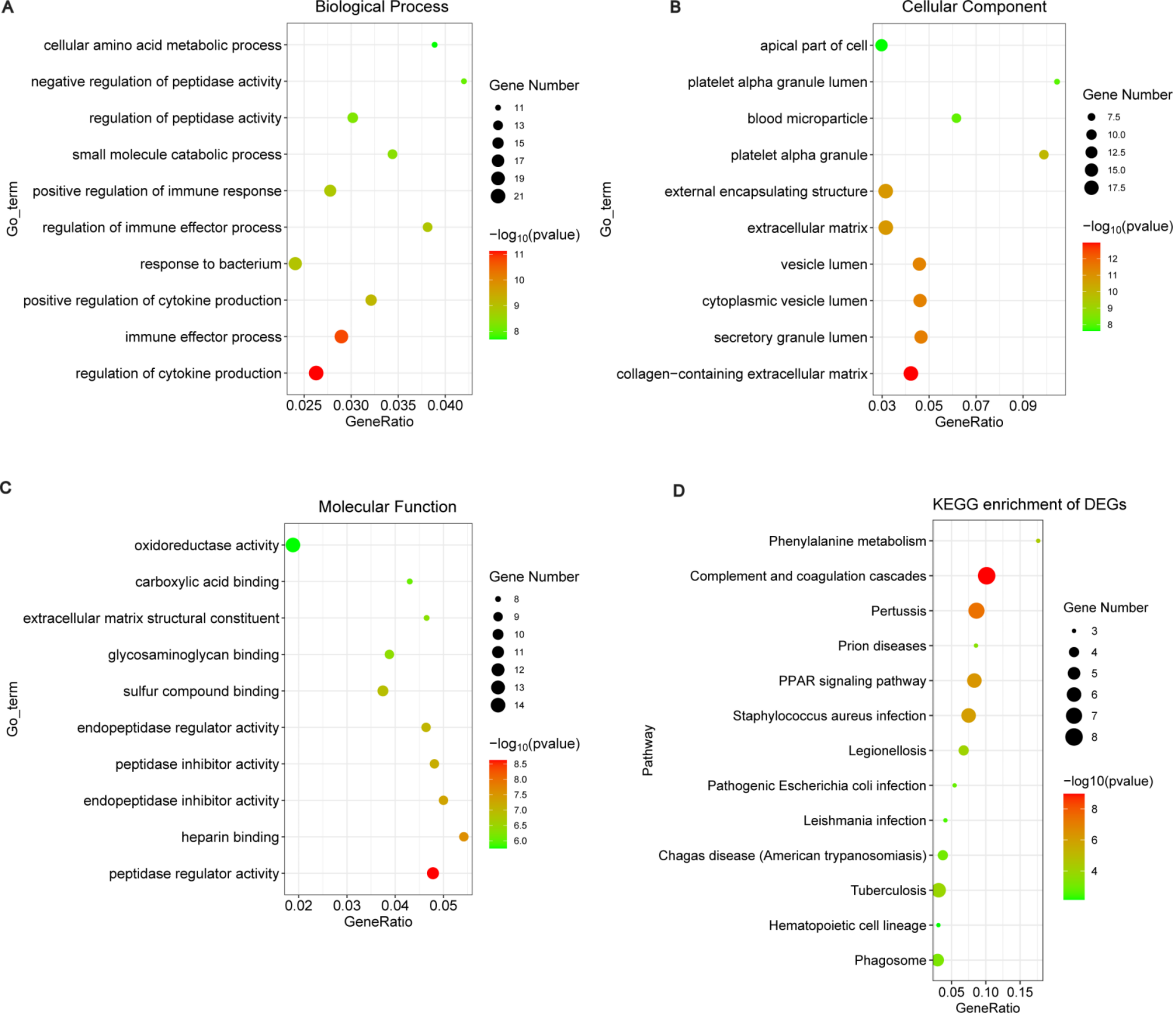
GO terms and KEGG pathways enrichment analyses of DEGs. **(A-C)** The bubble diagram of GO terms enrichment analyse. **(A)** BP terms. **(B)** CC terms. **(C)** MF terms. The *x*-axis indicates the gene ratio and the y-axis indicates GO terms. Distinct point shapes indicate distinct different categories and bubble size indicates gene count. Coloring indicates -log_10_(p value) with higher in red and lower in green. **(D)**The bubble plot of KEGG pathway analyse [18–20]. The x‐axis represents gene ratio and the y‐axis represents KEGG pathway. Bubble size indicates gene count and color indicates –log_10_(p value) with higher in red and lower in green

To elucidate the biological function of the screened DEGs, GO term and KEGG pathway enrichment analysis were conducted by Metascape. The GO enrichment analysis revealed that most significant enrichment in molecular function involved peptidase regulator activity, heparin binding and endopeptidase inhibitor activity. Most remark enrichment in biological process consisted of regulation of cytokine production, immune effector process and response to bacterium. Changes in cell component of DEGs were mainly enriched in collagen-containing extracellular matrix, secretory granule lumen and cytoplasmic vesicle lumen (Fig. 3A-C). KEGG signaling pathways results showed that DEGs mainly focused on complement and coagulation cascades, pertussis and PPAR signaling pathway (Fig. 3D).

### Functional enrichment analysis of all detected genes

**Fig. 4:**
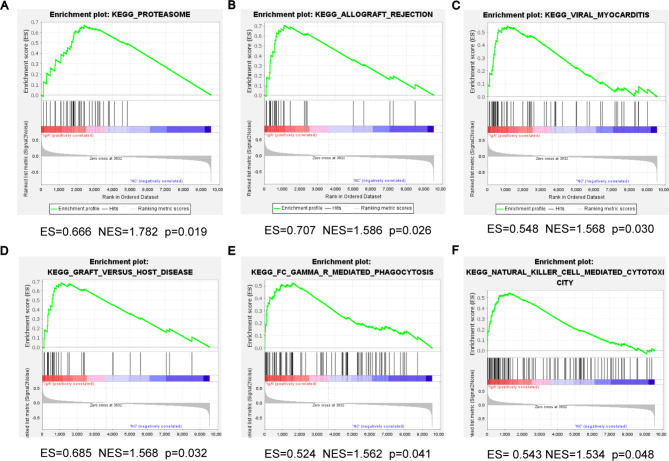
GSEA analysis demonstrating most enriched gene sets between the healthy and IgAN group. **(A)** The most significant enriched gene set was proteasome pathway (ES = 0.666, NES = 1.782, p < 0.05). **(B)** The second significant enriched gene set was allograft rejection (ES = 0.707, NES = 1.586, p < 0.05). **(C)** The third significant enriched gene set was viral myocarditis (ES = 0.548, NES = 1.568, p < 0.05). **(D)** The fourth significant enriched gene set was graft versus host disease (ES = 0.685, NES = 1.568, p < 0.05). **(E)** The fifth significant enriched gene set was FCγR mediated phagocytosis (ES = 0.524, NES = 1.562, p < 0.05). **(F)** The sixth significant enriched gene set was natural killer cell mediated cytotoxicity. (ES = 0.543, NES = 1.534, p < 0.05). GSEA: gene set enrichment analysis; ES: enrichment score; NES: normalized enrichment score; IgAN: IgA nephropathy

GSEA analysis was also performed to estimate the key pathways correlated with the IgAN group. The results suggested that most of the enriched gene sets were involved in proteasome pathway at the screening criteria of p < 0.05 and FDR < 0.25 (Fig. 4A). When the screening criterion for significant gene sets was p < 0.05, the most significantly enriched pathways included proteasome, allograft rejection, viral myocarditis, graft versus host disease, FCγR mediated phagocytosis, and natural killer cell-mediated cytotoxicity (Fig. 4A-F).

### PPI network constitution of DEGs and hub genes recognition

**Fig. 5:**
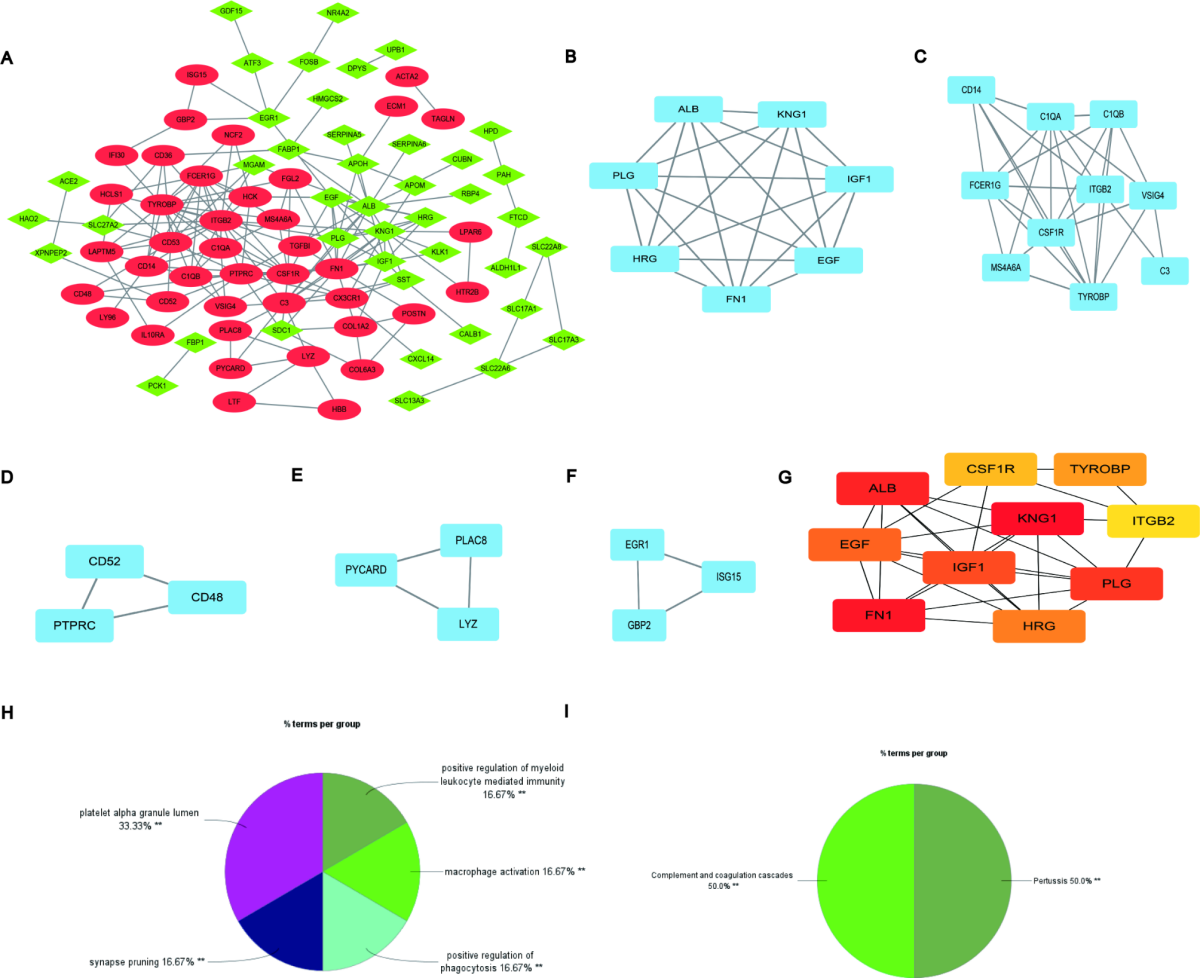
PPI network of DEGs, five cluster modules extracted by MCODE and identification of hub genes. **(A)** A network of PPI among the DEGs was established on STRING database. The highly expressed genes are illustrated by the red ellipses and lowly expressed genes by the green diamonds. Nodes represent genes, while edges represent protein-protein interaction. **(B)** Cluster 1 (MCODE score = 7). **(C)** Cluster 2 (MCODE score = 6.667). **(D)** Cluster 3 (MCODE score = 3). **(E)** Cluster 4 (MCODE score = 3). **(F)** Cluster 5 (MCODE score = 3). **(G)** Ten crucial genes were screened through MCC algorithm in Cytoscape. The higher the score, the deeper the color. **(H)** The GO terms enrichment analysis of the ten identified hub genes. **(I)** KEGG pathways analysis of identified ten hub genes. DEG: differentially expressed gene; PPI: protein-protein interaction

To investigate the interaction between proteins encoded by DEGs, PPI network was constructed by STRING tools and visualized by Cytoscape (Fig. 5A). The network was comprised of 83 nodes and 178 edges. And the down- and up-regulated proteins were represented by green diamond and red ellipse, respectively. In addition, the five most significant modules were detected by MCODE plugin in Cytoscape. As shown in Fig. 5B-F, cluster 1 possessed the highest cluster score (score: 7, 7 nodes and 21 edges), which was followed by cluster 2 (score: 6.667, 10 nodes and 30 edges), cluster 3 (score: 3, 3 nodes and 3 edges), cluster 4 (score: 3, 3 nodes and 3 edges), and cluster 5 (score: 3, 3 nodes and 3 edges). In total, 26 hub genes were screened from these five key modules. Subsequently, the top 10 hub genes (KNG1, FN1, ALB, PLG, IGF1, EGF, HRG, TYROBP, CSF1R, ITGB2) were extracted with CytoHubba (Fig. 5G). Later, our study took the intersection of these hub genes obtained by the two methods as the final hub genes and listed them in Table 2. Within ClueGO and Cluepedia, GO and KEGG enrichment analysis of these 10 final hub genes revealed that they were significantly enriched in platelet alpha granule lumen, synapse pruning, positive regulation of myeloid leukocyte mediated immunity, positive regulation of phagocytosis, macrophage activation, complement and coagulation cascades, and pertussis (Fig. 5H-I).

**Table 2:** Ten hub genes identified via Cytohubba

Gene symbol	Description	logFC
KNG1	kininogen 1	-1.50
FN1	fibronectin 1	1.12
ALB	albumin	-2.44
PLG	plasminogen	-1.43
IGF1	Insulin like growth factor 1	-1.10
EGF	epidermal growth factor	-1.33
HRG	histidine rich glycoprotein	-1.06
TYROBP	transmembrane immune signaling adaptor TYROBP	1.70
CSF1R	colony stimulating factor 1 receptor	1.01
ITGB2	Integrin subunit beta 2	1.30

### The interaction between hub genes and IgAN based on the CTD database

**Fig. 6:**
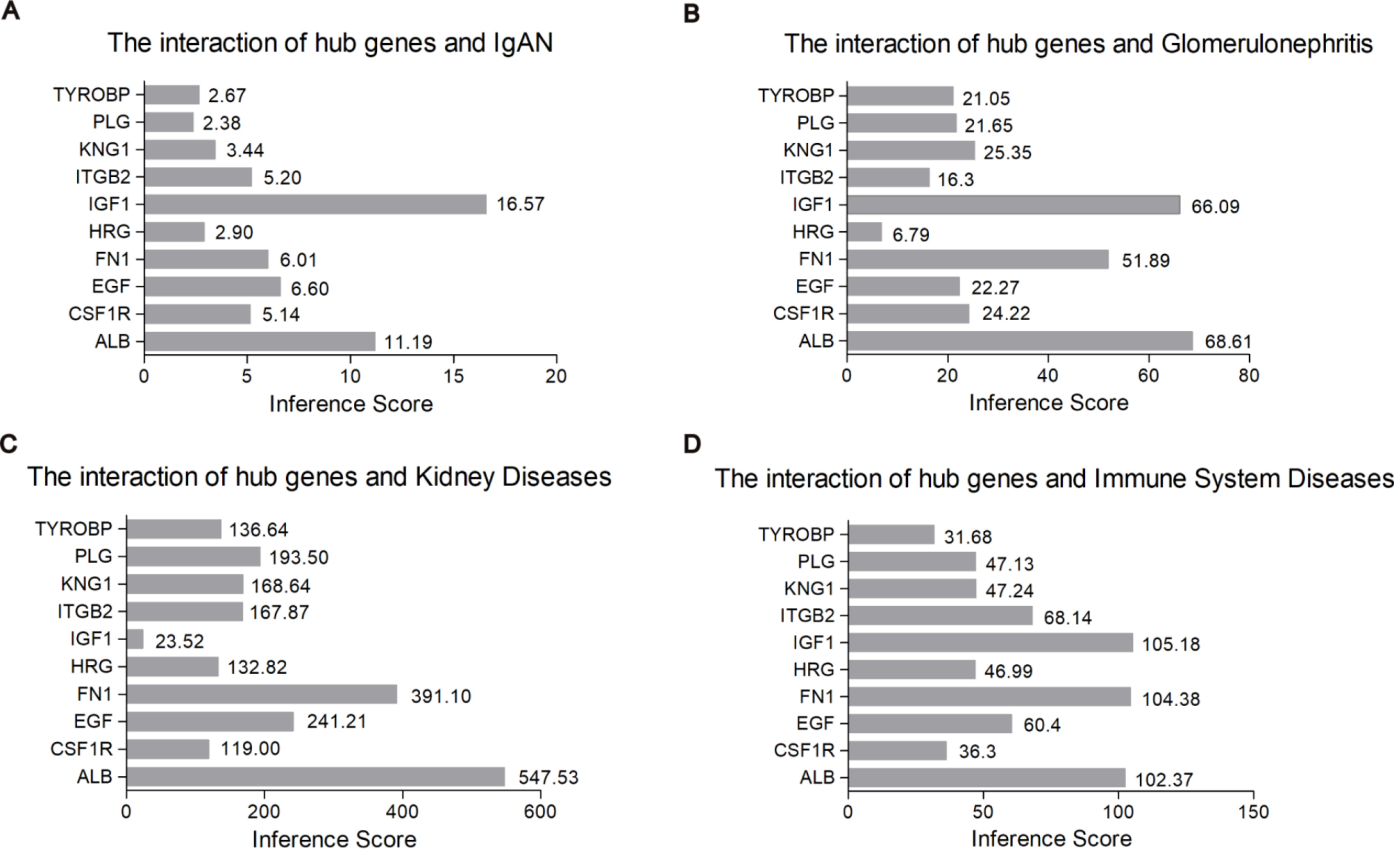
Recognization of potential crucial genes related to IgAN by CTD database

To identify candidate crucial genes interrelated with IgAN, the CTD database was employed to evaluate the relationship between selected hub genes and IgAN. As shown in Fig. 6, 10 hub genes targeting IgAN, glomerulonephritis, kidney diseases and immune system diseases. Inference scores embodied the correlation between chemicals, disease and genes. Apparently, ALB, IGF1 and FN1 were highly interconnected with IgAN.

### Immune cell infiltration in IgAN

**Fig. 7:**
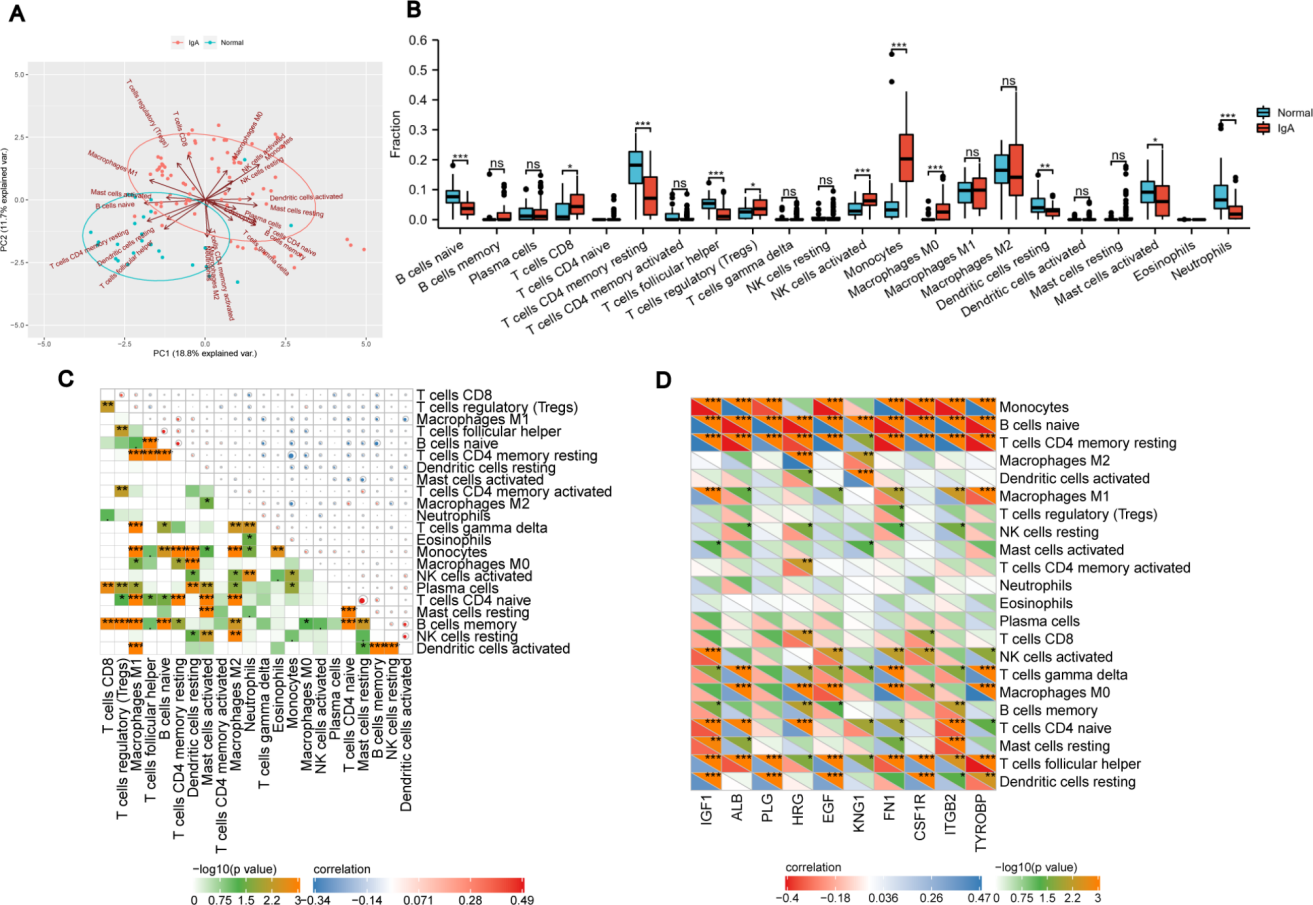
The immune landscape in IgAN and normal controls. **(A)** Principal components analyses (PCA) performed on all samples. **(B)** The difference in infiltrating immune cells between IgAN and the normal group. The IgAN group was illustrated in red color and the normal group was illustrated in blue color. **(C)** Correlation heatmap of all 22 immune cells. The size of the colored dots indicates the strength of the correlation. The red color stands for a positive correlation, while the blue color stands for a negative correlation. Darker color indicates a stronger correlation. *p < 0.05, **p < 0.01, ***p < 0.001. **(D)** Correlation heatmap between hub genes and immune cells infiltration. The color depth of the triangle below is positively correlated with the correlation coefficient. The red color indicates a negative correlation, while the blue color indicates a positive correlation. The color depth of the upper triangle indicates p value. *p < 0.05, **p < 0.01, ***p < 0.001

It is well known that IgAN is determined synthetically by genetic factors, environmental disturbance and immune response. Among them, immune inflammation achieved a dominant position in the occurrence and progression of IgAN. PCA cluster analysis is a tool to evaluate the consistency of biological duplication and divergence among distinct populations. So this PCA cluster plot results indicated that immune infiltration differed substantially between IgAN patients and the healthy group (Fig. 7A). Then, our study investigated the distribution of 22 types of immune cell subtypes in tissue using the CIBERSORT algorithm. From the box diagram in Fig. 7B, it was found activated NK cells, monocytes and M0 macrophages, CD8 + T cells, regulatory T cells infiltrated more, while naïve B cells, resting CD4 memory T cells, T cells follicular helper, resting dendritic cells, activated mast cells, and neutrophils infiltrated less in glomerular tissue of IgAN patients compared to that of normal group. Furthermore, correlation heatmap confirmed that monocytes were significantly positively correlated with M0 macrophages. Memory B cells and naive CD4 + T cells also showed a positive relationship significantly. However, resting CD4 memory T cells were significantly negatively correlated with monocytes. Naive B cells and memory B cells also had a great negative correlation. And there was a high correlation between activated mast cells and resting mast cells (Fig. 7C). The correlation heat map was performed to demonstrate the correlation of identified hub genes with immune cell infiltration. The results revealed that IGF1, EGF, HRG, FN1, ITGB2 and TYROBP were highly associated with the abundance of monocytes, naive B cells, resting CD4 memory T cells, and T cells follicular helper, which provided insight into the potential role of these crucial genes in immune landscape (Fig. 7D).

### mRNA expression of hub genes in renal glomeruli of IgAN Patients Using nephroseq v5 online platform

**Fig. 8:**
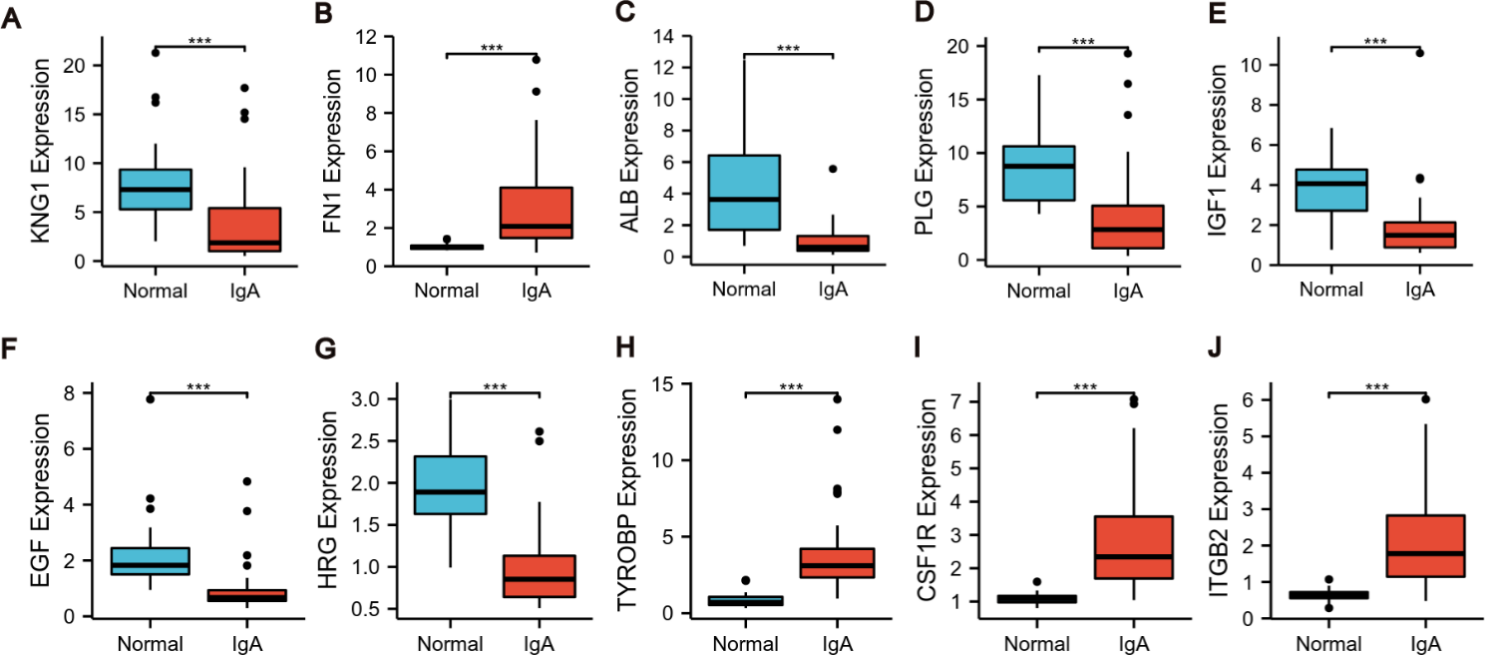
mRNA expression of hub genes in the glomerulus of IgAN patients based on Nephroseqv5 platform. **(A)** The expression of KNG1 decreased in IgAN. **(B)** The expression of FN1 elevated in IgAN. **(C)** The expression of ALB downregulated in IgAN. **(D)** The expression of PLG reduced in IgAN. **(E)** The expression of IGF1 decreased in IgAN. **(F)** The expression of EGF descended in IgAN. **(G)** The expression of HRG declined in IgAN. **(H)** The expression of TYROBP upregulated in IgAN. **(I)** The expression of CSF1R enhanced in IgAN. **(J)** The expression of ITGB2 increased in IgAN. p < 0.05 was considered statistically significant. * p < 0.05, **p < 0.01, ***p < 0.001. IgAN: IgA nephropathy; mRNA: messenger RNA

Comparing with the normal group, the expression of FN1, TYROBP, CSF1R, and ITGB2 significantly increased while that of other 6 hub genes visibly decreased in the IgAN samples (Fig. 8).

### ROC curve analysis of 10 hub genes in predicting IgAN

**Fig. 9:**
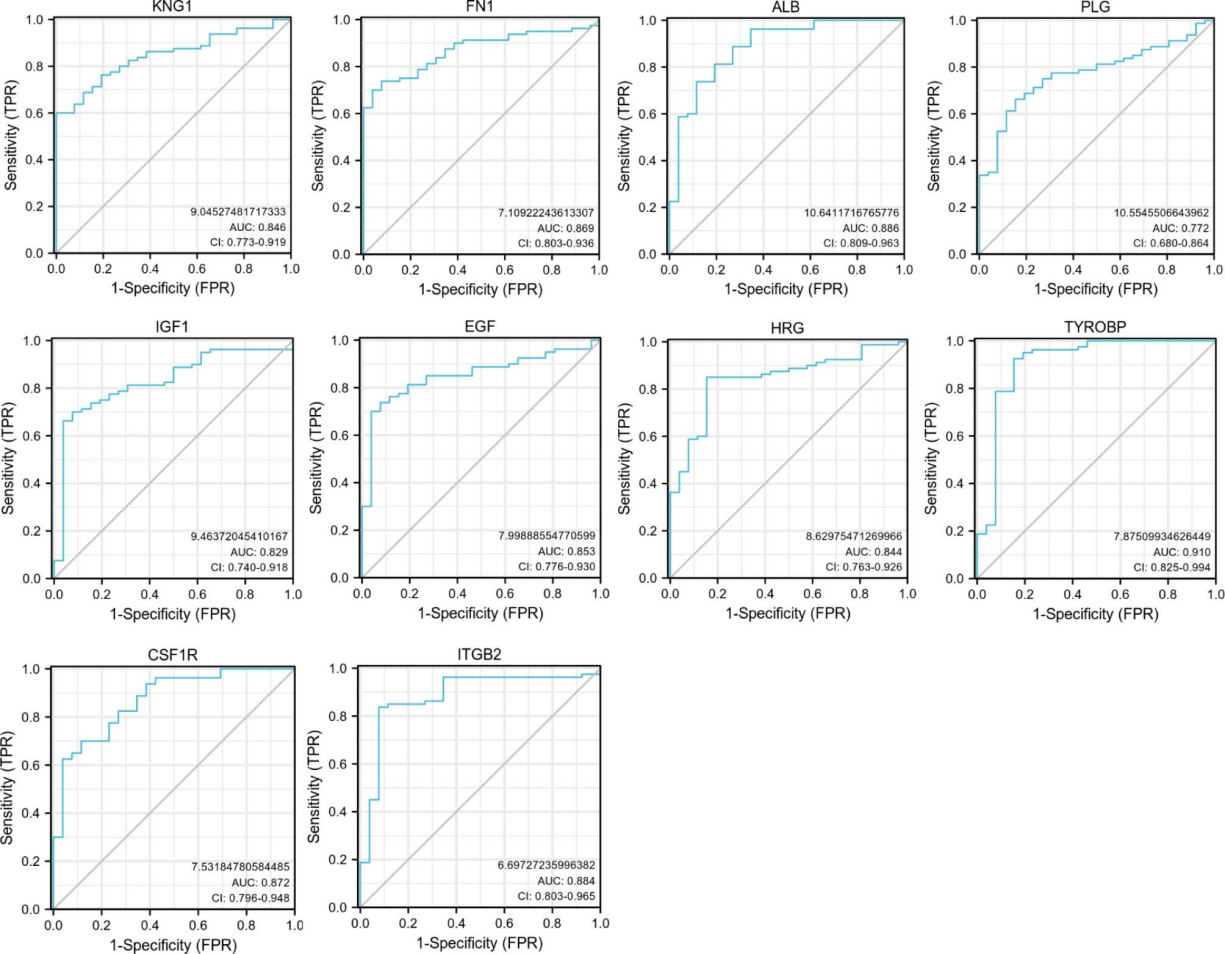
Diagnostic accuracy of hub genes. ROC: receiver operating characteristic; AUC: area under the ROC curve

The receiver operating characteristic *(*ROC*)* curve, a fundamental tool for diagnostic test evaluation, was brought to explore the diagnostic value of identified hub genes, of which area under the curve (AUC) served as a quantitative indicator for intrinsic effectiveness of diagnostic test. According to Fig. 9, TYROBP has the strongest diagnostic ability for IgAN with the highest AUC value of 0.910, which was followed by ALB (AUC: 0.886) and ITGB2 (AUC: 0.884). The predictive value of other genes in the diagnosis of IgAN are follows: CSF1R (AUC: 0.872), FN1 (AUC: 0.869), EGF (AUC: 0.853), KNG1 (AUC: 0.846), HRG (AUC: 0.844), IGF1 (AUC: 0.829), PLG (AUC: 0.772). Therefore, TYROBP, ALB and ITGB2 may serve as novel diagnostic biomarkers for IgAN.

### Identification of small molecule therapeutic drugs to arrest glomerular injury of IgAN

**Fig. 10:**
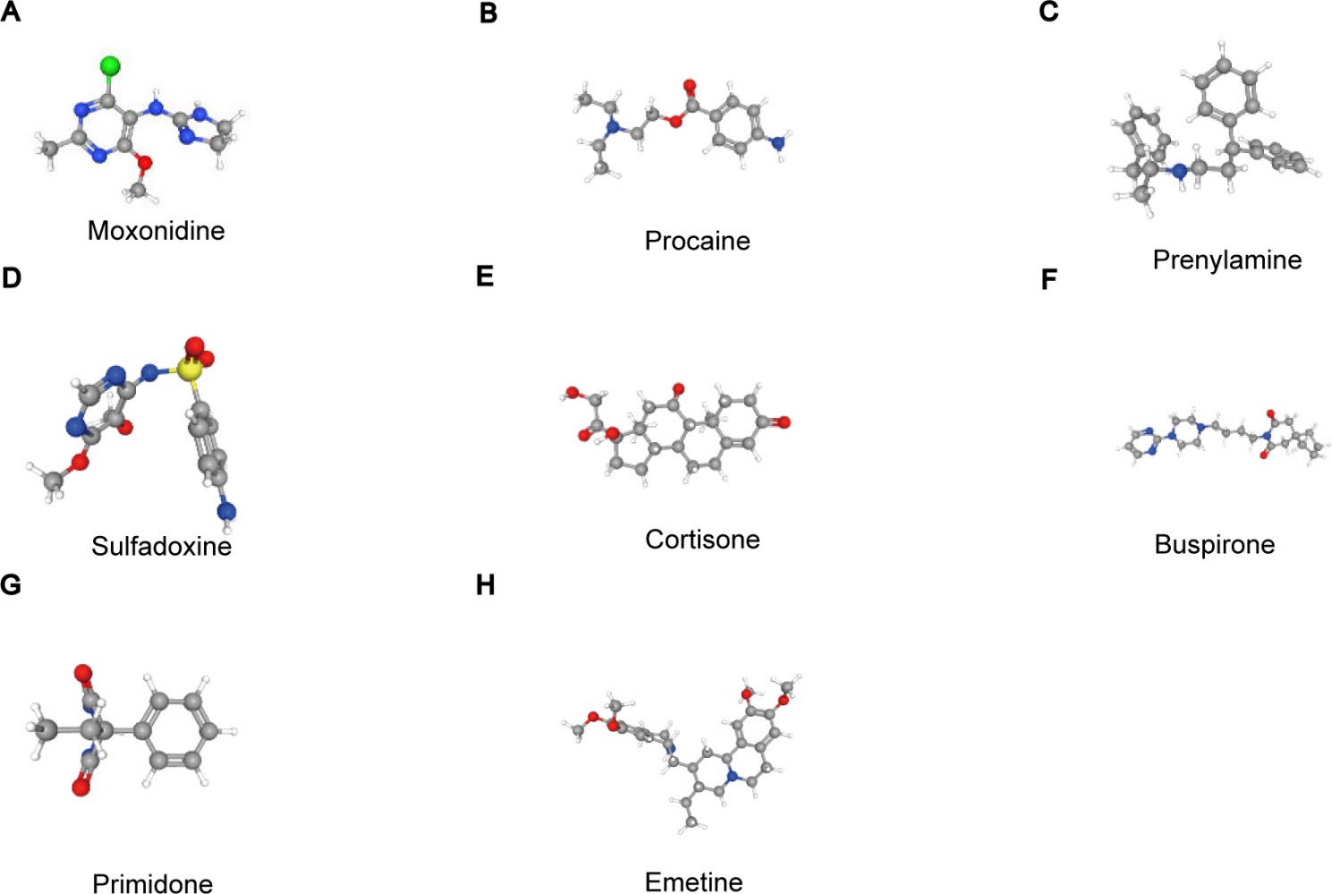
Ten most significant small molecules as potential drugs for IgAN treatment targetting ten hub genes. **(A–H)** Predicted chemical structure of targeted drugs. IgAN: IgA nephropathy

Aiming at predicting potential therapeutic agents targeting IgAN, CMap database was applied to acquire small molecular compounds which may reverse hub genes expression. The top 10 small molecular compounds were screened (Table 3). Among them, verteporfin, moxonidine, procaine, and prenylamine were the most significant four compounds, indicating that these compounds may become candidate drugs to improve IgAN. Further, the PubChem database, a public repository for information on small molecules and their biological activities, was utilized to acquire the chemical structures of small molecular compounds among which the structures of verteporfin and STOCK1N-35,215 had not been identified (Fig. 10).

**Table 3:** Potential drugs were provided by CMap database according to hub genes

Cmap name	Mean	N	Enrichment	*p*	Percent non-null
verteporfin	-0.859	3	-0.99	0	100
moxonidine	-0.646	3	-0.896	0.00218	100
procaine	-0.597	5	-0.848	0.00024	100
prenylamine	-0.614	4	-0.814	0.00225	100
sulfadoxine	-0.561	3	-0.795	0.01757	100
cortisone	-0.532	3	-0.794	0.01785	100
buspirone	-0.525	4	-0.778	0.00501	100
STOCK1N-35,215	-0.553	3	-0.768	0.02552	100
primidone	-0.251	4	-0.699	0.01725	50
emetine	-0.454	4	-0.687	0.02067	75

### The validation of TYROBP in IgAN

**Fig. 11:**
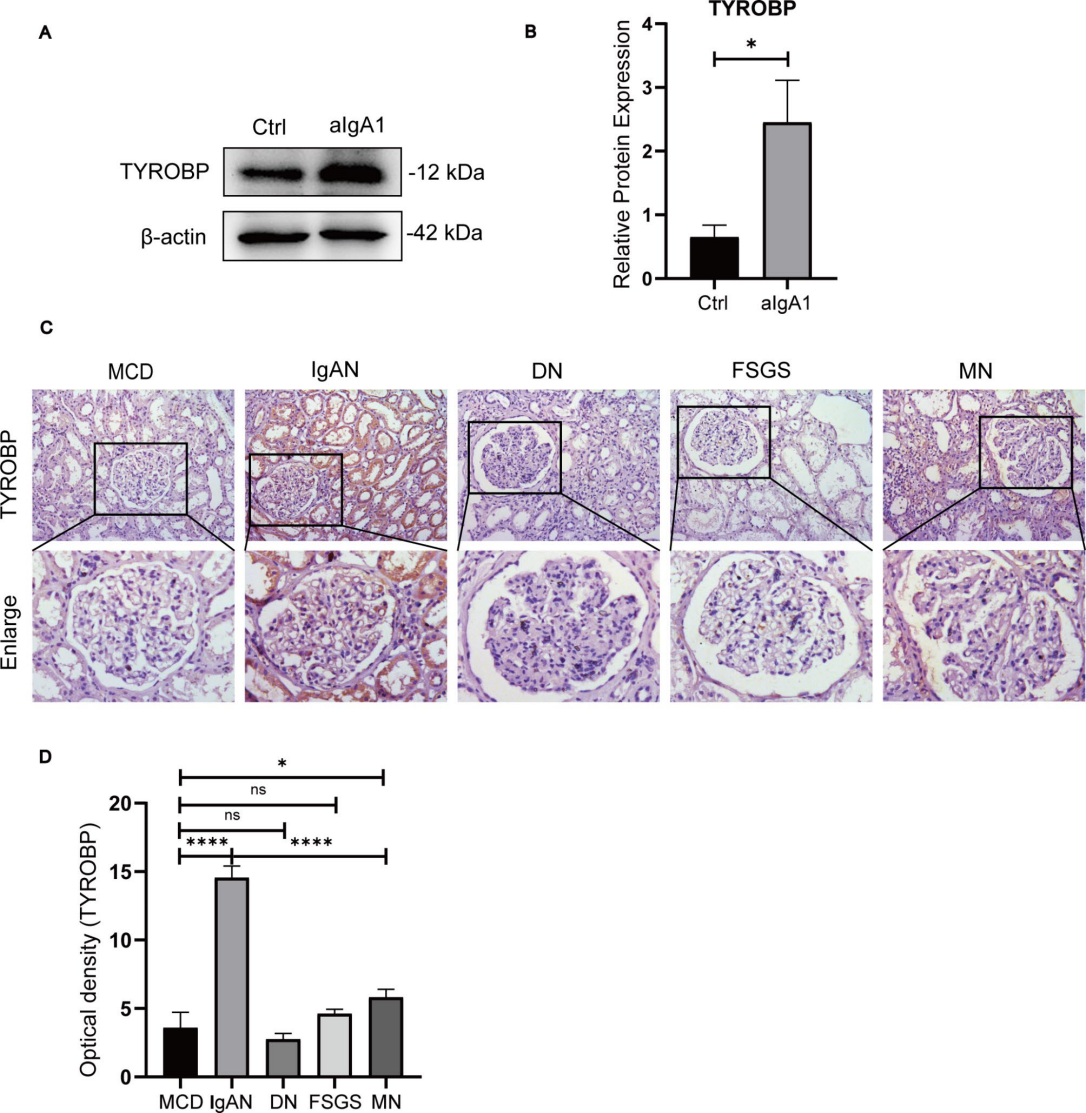
The expression level and diagnostic significance of TYROBP were verified. **(A)** TYROBP protein expression was measured through western blotting. **(B)** Quantitative analysis indicated that the expression of TYROBP in aIgA1-stimulated HMCs was evidently up-regulated compared with the control group. **(C)** Immunohistochemical staining for TYROBP was conducted in different renal pathologies to evaluate its diagnostic accuracy in IgAN. Macroscopic and microscopic examination were presented. **(D)** Quantitative results of immunohistochemical staining revealed that TYROBP was markedly higher in IgAN than that in other renal pathologies. *p < 0.05, **p < 0.01, ***p < 0.001, ****p < 0.0001. Data are representative of 3 independent experiments and are expressed as mean ± SD

Based on the highest expression and the highest diagnostic value as well as the close correlation with immune infiltration among these five unexplored hub genes, TYROBP was further validated in vivo and in vitro. The results showed that TYROBP was highly expressed in aIgA1-treated HMC and renal tissues from IgAN patients (Fig. 11). However, TYROBP was expressed at low abundance in other types of kidney disease including minimal change disease (MCD), diabetic nephropathy (DN), focal segmental glomerular sclerosis (FSGS) and membranous nephropathy (MN) (Fig. 11). These results demonstrated that TYROBP possessed considerable diagnostic value for IgAN.

## Discussion

IgAN is the most common cause of primary glomerulonephritis around the world, which comprises a large fraction of the ESRD (20–40%) [3, 4, 28]. Because of high morbidity and mortality rates, it has brought about an immense social and economic burden [3]. Nevertheless, the pathophysiologic mechanisms of IgAN have not been elucidated thoroughly. Invasive renal biopsy is the only currently available tool to identify IgAN and current treatment methods have not achieved the desired efficacy in the clinic. Thus, there is a pressing need for developing novel non-invasive approaches and more effective therapeutic options based on a full understanding of pathogenesis.

Our study merged and analyzed gene expression profiling of 80 IgAN samples and 27 normal samples from 3 datasets by bioinformatics methods. As a result, 113 DEGs were identified, consisting of 49 genes up-regulated and 64 down-regulated. Of these, 67 tissue/organ-specific expressed genes were screened via BioGPS and the results indicated that the haematologic/immune system held the highest degree of specificity, which could clarify and verify the autoimmune nature of IgAN [29, 30]. Meanwhile, second organ-specific expressed system was the digestive system consistent with the common occurrence of intestinal mucosal infections in patients with IgAN [31]. Evidence emerged that mucosal immune reaction, particularly in the intestines, may be the major source of hypo-galactosylated IgA1 [32]. Furthermore, quite a few genes were not only highly expressed in kidney, but were also abundantly expressed in digestive system. Combining the hypothesis of gut-kidney axis existing in IgAN, there was an intricate crosstalk between gut and kidney. Various pathogenic virulence incited the injury of intestinal mucosal barrier and thus promoted the enhanced production of aberrantly glycosylated polymeric IgA1, which was then released into the blood circulation and deposited in the glomeruli [6]. Further, this validated the fact that the kidney merely acted as an innocent bystander. GO annotation analysis of DEGs suggested that collagen-containing extracellular matrix, regulation of cytokine production, and immune effector process were mainly enriched, which are very consistent with the characteristics of IgAN including deposited circulating immune complexes-induced proliferation of glomerular cells and overproduction of extracellular matrix (ECM), secretion of inflammatory cytokines, as well as infiltration of diverse immune cells [3, 33–35]. Additionally, KEGG pathways result showed that the prominent pathways were enriched in complement and coagulation cascades, pertussis and PPAR signaling pathway, confirming that immune responses and inflammation exerted great effects on the onset and development of IgAN. It has been reported that toll-like receptor 4 (TLR4)-induced proinflammatory effects, which could accelerate the progression of IgAN, can be efficiently attenuated by PPAR-γ agonists in vivo and in vitro [36, 37]. Further, GSEA software was employed to conduct KEGG pathway analysis targeting a total of 9556 genes and the results suggested the IgAN group was most positively associated with proteasome pathway. The switch from proteasome to immunoproteasome was observed in those with ongoing progressive IgAN, which represents the hyperactivation of the proteasome system responding to infectious agents-induced IFNs production or oxidative stress [38]. A single-center open-label pilot trial implicated that bortezomib, a proteasome inhibitor targeting plasma cells, achieved more expected efficacy in patients with severe IgAN compared to rituximab targeting B-cell depletion [39]. Then, PPI network was constructed to distinguish 10 hub genes, including KNG1, FN1, ALB, PLG, IGF1, EGF, HRG, TYROBP, CSF1R, and ITGB2. The results from enrichment analysis of these hub genes demonstrated that they were highly correlated with immune-related pathways.

Among these hub genes, five genes, namely FN1, ALB, PLG, IGF1 and EGF, have been reported in IgAN. FN1 is identified as the principal component of ECM, and elevated FN1 levels reflect more severe renal fibrosis in IgAN [40]. Roszkowska Blaim et al. found that urinary excretion of FN may be an indicator to evaluate the disease activity of IgAN [41]. Consistent with these studies, our study found that FN1 expression was enhanced in IgAN, which may indicate a higher degree of fibrosis. ALB encodes serum albumin. As the most abundant protein in blood, serum albumin manifests the nutrition status and regulates the plasma colloid osmotic pressure and functions as a transporter of numerous substances. Some data revealed that time-averaged serum albumin (TA-ALB) may be predictive of long-term outcome in IgAN patients who have achieved remission [42]. Compared with the control samples, the ALB expression was markedly decreased in IgAN patients, which may be reflective of a poor prognosis. PLG (Plasminogenis), a serine protease that circulates in blood plasma as an inactive zymogen, is converted to the active protease, plasmin. Plasmin degrades many blood plasma proteins, containing fibrin-containing blood clots. Accumulating evidence showed that a considerable proportion of IgAN patients presented stable fibrin deposition followed by fibrinolysis and platelet viability in the glomerulus [43]. Our study suggested PLG was greatly down-regulated in patients with IgAN, which may mirror PLG was over activated and the activity of fibrinolytic system was enhanced. IGF1 has been proved significant in fostering mesangial cells and podocytes proliferation and ECM reshaping [44]. In addition, most of IgAN patients showed increased IGF-I expression in peripheral blood mononuclear cells (PBMC), while not detected in patients with other glomerulonephritis or normal samples [45, 46]. It was also found that the level of IGF-1 in PBMC had a strong positive correlation with urinary protein excretion and histopathological alterations [46]. Thus, these evidences illustrated that abnormal expression of IGF-I in PBMC may act as a useful biomarker of IgAN activity and progression. Furthermore, the rs2195239, rs1520220, and rs978458 variants played vital roles in the pathological progression of IgAN [47]. Compared to the healthy group, the expression of IGF1 in IgAN was slightly lower in our study, which calls for further investigation. In IgAN patients, urinary IL-6 and MCP-1 exhibited a significant increase, while EGF excretion exhibited a reduction. These changes paralleled the progression of staging. Previous studies have indicated that EGF urinary excretion or 24-hours excretion of EGF may be an effective indicator to estimate interstitial fibrosis and renal function outcomes in patients with IgAN [48]. Further evidence suggested urinary IL-6/EGF ratio might serve as a prognostic biomarker for renal impairment and EGF/MCP-1 ratio in urine may function as a valuable measure to predict the prognosis of ESRD in IgAN patients [49, 50]. In our study, EGF showed significantly lower levels in IgAN patients than in healthy individuals. These may reflect the progression and prognosis of IgAN.

Thus far, no study has been performed on the correlation between such hub genes and IgAN as KNG1, HRG, TYROBP, CSF1R and ITGB2. Kininogen 1 (KNG1) could be degraded to High-molecular weight kininogen (HMWK) and low-molecular weight kininogen (LMWK), and HMWK played a significant role in inflammation and coagulation. Some data demonstrated that KNG1 rs5030062 and rs710446 variants were bound up with higher eGFR [51]. Our study found that KNG1 was dramatically down-regulated in IgAN samples compared to normal samples, which may indicate increased degradation. Being an adapter, histidine-rich glycoprotein (HRG) participates in a wide variety of pathways, such as inflammation, immune function, fibrinolysis, and coagulation [52–54]. As was reported that HRG could prevent septic lethality via inhibiting neutrophil adhesion to endothelial cells, indicating that HRG may become a therapeutic option for inflammatory diseases [55]. Brier et al. illustrated that HRG significantly improved risk prediction for AKI following cardiac surgery [56]. At present, HRG has not been reported in IgAN-related studies. TYROBP, named as TYRO protein tyrosine kinase binding protein, is an adapter protein containing an immunoreceptor tyrosine-based activation motif. It involves in diverse physiological processes including signal transduction, cell activation, immune function, immune inflammation, and apoptosis through non-covalently associating with receptors on the surface of various immune cells. The abnormal expression of TYROBP has been reported to take part in numerous diseases, such as Alzheimer’s disease, breast cancer, osteosarcoma and renal cell carcinoma. Takahashi et al. found that TYROBP improved microglia phagocytosis of amyloid β and gave rise to the occurrence and development of AD [57]. Pottier et al. considered that TYROBP could be used for early diagnosis and intervention of AD [58]. Wang et al. and Li F et al. indicated that in renal cell carcinoma, TYROBP was significantly increased and was related to poor prognosis [59, 60]. Nevertheless, TYROBP has not been reported in IgAN. The findings indicated that TYROBP expression was greatly enhanced in IgAN samples compared with the normal samples. Notably, TYROBP held a significant diagnostic value for IgAN, as indicated by the AUC up to 0.910. Thus, TYROBP was considered as a novel and promising index for the diagnosis of IgAN. CSF1R gene-encoded protein is the receptor for colony stimulating factor 1, which is related to the production, differentiation, as well as function of macrophages. CSF1R was predominantly expressed in immune cells, such as monocyte, macrophage, bone marrow cell precursors, and microglia in the central nervous system [61]. Notably, CSF-1 can induce tubule proliferation, which is critical for kidney repair from acute kidney injury. Perry et al. demonstrated that enhanced CSF-1 expression in tubule epithelial cells protected against acute kidney injury through binding to its receptor CSF1R and promoting tubular cell proliferation [62]. Proximal tubule-specific knockout of CSF-1 led to a significant increase in neutrophils and a decrease in macrophages/dendritic cells [62]. In our study, the expression of CSF1R was elevated in glomeruli of IgAN patients, which may be responsible for immune cell infiltration, particularly macrophages. Integrin subunit beta 2(ITGB2) encodes the β integrin subunit, which can constitute distinct integrins through interacting with various α subunits. It was reported that ITGB2 can foster the adhesion of leukocytes to the vascular endothelium and subsequent extravasation [63, 64]. ITGB2 was also found related to ECM-remodelling, which was correlated with poor survival outcomes in patients with RCC [65]. In CKD patients, ITGB2 was negatively associated with estimated glomerular filtration rate (eGFR). Additionally, bioinformatics analysis illuminated that other diseases which were associated with ITGB2 included lupus nephritis and diabetic nephropathy. In this study, ITGB2 was identified highly expressed in patients with IgAN, which might be associated with immune cell infiltration, renal fibrosis and decreased renal function.

The result from the CTD database showed that ALB, IGF1 and FN1 exhibited a higher score with IgAN, reflecting a tight linkage between these three crucial genes and the occurrence and development of IgAN. Even further, ROC curve analyses were conducted to confirm the sensitivity and specificity of hub genes for IgAN diagnosis. As the results demonstrated, all the hub genes have good diagnostic performance with an AUC of over 0.75. Especially TYROBP, holding the highest AUC value of 0.910, may serve as a potential molecular biomarker for the diagnosis of IgAN.

It has been widely acknowledged that constant Gd-IgA1 deposition brings about the proliferation of mesangial cells and subsequent production of cytokines and chemokines, which act as the key mediators of the crosstalk between pathological mesangium and other renal cells [66]. As a result, glomerulosclerosis and tubulointerstitial fibrosis are caused. Notably, growing evidence suggests infiltrating immune cells in the kidney play a critical role in the pathogenesis of IgAN [34, 67, 68]. Hence, CIBERSORT was employed to quantify the immune infiltration in IgAN patients for investigating the role of immune cells in IgAN. It was found that activated NK cells, monocytes, M0 macrophages, CD8 + T cells, and regulatory T cells were considerably increased in the glomerular tissue of IgAN, while naïve B cells, resting CD4 memory T cells, T cells follicular helper, resting dendritic cells, activated mast cells, and neutrophils memorably decreased. Previous studies demonstrated that HLA-DR expressing NK cells were prominently augmented in IgAN patients and patients with higher HLA-DR manifested faster deterioration of renal function [69]. The CD56dimCD16 + subpopulation accounts for 90% of NK lymphocytes and has higher cytotoxicity and a higher capacity for cytokine secretion. Esteve Cols C et al. stated that CD56dimCD16 + NK cells were present in higher proportions in IgAN patients compared with the normal [70]. Consequently, NK cells may involve in IgAN pathogenesis via creating an inflammatory microenvironment and attracting more immune cells. It has been proved that infiltrating immune cells in the kidney, especially monocytes/macrophages, play an important role in albuminuria and kidney impairment in IgAN. Elevated Tim-3 monocytes/macrophages in circulation and renal tissue might contribute to the pathogenesis of IgAN and could be used to evaluate disease severity [71]. Increasing evidence indicated an association between the abundance of renal macrophages and the severity of IgAN [72]. More recently, two single-cell RNA-sequencing studies in IgAN confirmed monocytes and macrophages greatly enhanced in the kidney of IgAN patients. Zheng et al. elucidated that PLGRKT and CCL2 were upregulated in mesangial cells and recruited monocytes/macrophages [34]. Tang et al. proved that three genes FAM49B, GPX3 and FCGBP associated with ROS production, mitochondrial function and EMT, respectively, were downgregulated in macrophages of IgAN patients [73]. In addition, Tomino et al. uncovered that CD8 + T cells were the most abundant glomerular-infiltrating cell in IgAN patients [74]. Anti-CD8 T cell treatment has been proved to trigger a profitable effect of suppressing mesangial expansion in an animal model of IgAN [75]. Another study showed that, compared to children with non-progressive IgAN, significantly higher percentages of CD8 + T cells in the glomeruli and in the interstitium were detected in the children with progressive IgAN [76]. Furthermore, Zheng et al. also revealed that genes associated with effector T cell marker and the cytotoxicity, were greatly decreased, while T cell exhaustion-related genes were increased in CD8 + cytotoxic T lymphocytes, indicating CD8 + cytotoxic T lymphocytes dysfunction may be linked to IgAN progression [34]. An Italian study detected aberrant methylation in CD4 + T cells from IgAN patients and this may give rise to impaired TCR signaling and attenuated T cell activation [77]. Neutrophil apoptosis could be triggered by immobilized IgA [78], which might explain a marked reduction in the number of neutrophils. The results of our study combined with the above literature evidence have elucidated that immune cell infiltration is closely associated with IgAN progression and the potential mechanisms should be further explored. Furthermore, correlation analysis between immune cells revealed that monocytes were significantly positively correlated with M0 macrophages. Memory B cells also exhibited a significant positive correlation with naive CD4 + T cells. However, monocytes were significantly negatively correlated with resting CD4 memory T cells. Naive B cells and memory B cells also had a great negative correlation. These evidences suggest that there is an intricate interaction between immune cells and the underlying mechanisms need to be further clarified. The correlation heat map revealed that six identified hub genes, namely IGF1, EGF, HRG, FN1, ITGB2 and TYROBP, were strongly related to the abundance of monocytes, naive B cells, resting CD4 memory T cells, and T cells follicular helper. Among them, TYROBP was negatively correlated with T cell and B cell infiltration, and positively correlated with dendritic cells and mast cell infiltration. All these associations provided insight into the potential role of these crucial genes in the occurrence and progression of IgAN.

Based on the CMap database, ten key genes were used to search for potential drugs for IgAN treatment and the top ten candidate drugs were identified. Among these drugs, only cortisone, which belongs to corticosteroids and acts as one of existing immunosuppressive options, has been extensively used in the clinic to treat IgAN. Verteporfin, a apharmacological inhibitor of YAP, was reported to inhibit fibrosis in many models of kidney diseases including AKI, DKD and UUO [79–81]. A recent study demonstrated that verteporfin could alleviate renal inflammation mediated by tubular repair after ischemia/reperfusion [82]. In addition, verteporfin produced protective effects against UUO-induced renal tubulointerstitial inflammation and fibrosis by suppressing the TGF-β1/Smad signaling pathway [83]. Feng et al. showed that verteporfin also prevented kidney fibrosis by impeding Wnt5a- and TGFβ1-mediated M2 macrophage polarization [84]. Another study reported that verteporfin administration or specific deletion of YAP (yes-associated protein) in renal proximal tubule cells apparently mitigated renal tubulointerstitial fibrosis in DKD mice [80]. Moxonidine is an inhibitor of central sympathetic outflow, which can act centrally to decrease sympathetic nervous activity [85]. Therefore, it is generally viewed as an antihypertensive drug. Tsutsuiet al. showed that moxonidine served as an agonist of α2/imidazoline Ι1-receptor and repressed the renal sympathetic nervous system activity, thereby protecting against acute ischemic kidney injury [86]. Hausberg et al. revealed that a low dose of moxonidine generated significant and sustained suppression of sympathetic outflow without any detrimental effect on hemodynamics, which may improve the outcome of ESRD patients [87]. Procaine was illustrated to mitigate nephrotoxicity caused by cisplatin by forming a less toxic complex with cisplatin [88]. Nevertheless, there is no more related study on kidney diseases. Prenylamine acts as a calcium antagonist and has been applied for treating angina pectoris [89]. In addition, prenylamine could induce cell apoptosis, thus becoming a candidate agent for carcinoma [90]. Combining with pyrimethamine, sulfadoxine can be used for treatment and prevention of chloroquine-resistant malaria [91]. Buspirone can activate the 5-HT1A receptor and belongs to non-benzodiazepine anxiolytics [92, 93]. Bioinformatics analysis showed that STOCK1N-35,215 may be a targeted drug for recurrent implantation failure [94]. However, the present studies regarding the effects of STOCK1N-35,215 are limited and further inquiry is called for. Primidone is considered an aromatic antiepileptic used for the treatment of partial, generalized and complex seizures. More recently, Riebeling et al. revealed that RIPK1 activation and RIPK1-driven cell death, playing a crucial role in hyperinflammatory diseases including renal ischemia-reperfusion injury (IRI), can be prevented by primidone, an effective and specific inhibitor of RIPK1 [95]. Currently, primidone is FDA-approved and it has the potential to become a promising candidate for treating inflammatory disorders. Emetine is applied for treating acute amoebic dysentery and parenteral amoebiasis, however, It has certain toxicity. In addition, It has been proved to promote the degradation of HIF-2α in clear cell renal cell carcinoma [96]. Therefore, this warrants further work to investigate the effect of those compounds in IgAN as well as the underlying mechanisms.

Among these five unexplored hub genes, TYROBP held the highest expression level and highest diagnostic efficiency. Therefore, the following experiment focused on investigating this hub gene. In vitro and in vivo experiments, the results confirmed the high abundance of TYROBP in IgAN. Significantly, TYROBP could differentiate IgAN from other renal pathologies well, which showed that TYROBP may be a good diagnostic marker for IgAN.

This study has, however, certain limitations. First, the sample size is not big enough. Second, further clinical researches and basic experiments are required to validate the analytical results and explore the molecular mechanisms underlying these results. Third, CIBERSORT analysis relies on the restricted genetic data which may diverge from heterotypic cell-cell interactions, phenotypic plasticity, or disease-induced disturbances. While CIBERSORT has significantly lower estimation bias compared with other methods, some kinds of immune cells might be systematically overestimated or underestimated.

## Conclusion

In total, 113 DEGs and 10 hub genes were selected on the strength of bioinformatics methods, which may involve in the pathogenesis of IgAN and become diagnostic biomarkers and therapeutic targets for the disease. In addition, this study predicted several candidate drugs that may ameliorate glomerular injury and improve IgAN outcomes. Furthermore, TYROBP may serve as a good biomarker for the diagnosis of IgAN. Collectively, our results provide a more in-depth insight into the occurrence and progression of IgAN. Nevertheless, further clinical and basic studies are required to elucidate biological functions of those genes in IgAN.

## Electronic supplementary material

Below is the link to the electronic supplementary material.

Supplementary Material 1

## Data Availability

The datasets employed in our study can be acquired in the GEO repository (https://www.ncbi.nlm.nih.gov/geo/). The accession numbers are GSE37460, GSE99339, and GSE104948.
