# Supplementary figures and images for: Identifying potential biomarkers for the diagnosis and treatment of IgA nephropathy based on bioinformatics analysis
Source: BMC Med Genomics. 2023 Mar 28;16:63. doi: 10.1186/s12920-023-01494-y (PMC10044383; doi:10.1186/s12920-023-01494-y)

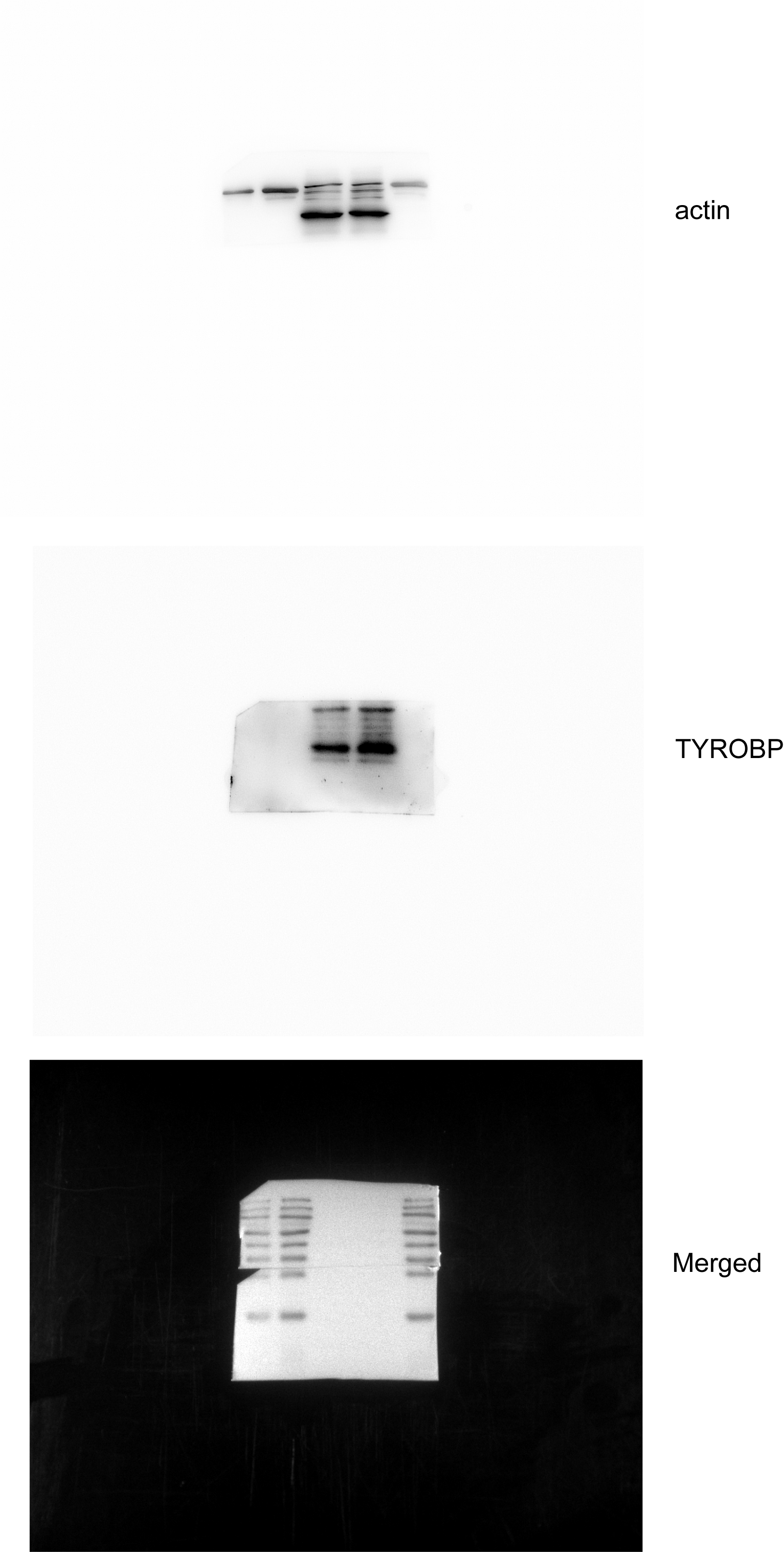

Supplement: Supplementary file 1 — Supplementary Material 1 [file 12920_2023_1494_MOESM1_ESM.png]
